# Maternal age and intracytoplasmic sperm injection outcome in infertile couples at Khartoum, Sudan

**DOI:** 10.12688/f1000research.7386.1

**Published:** 2015-11-24

**Authors:** Mohamed Ahmed, Osama Shareef, Ishag Adam, Duria Rayis

**Affiliations:** 1 Department of Obstetrics and Gynecology, Faculty of Medicine, University of Khartoum, Khartoum, 11111, Sudan; 2 Adam and Hawa Fertility Center, Khartoum, 11111, Sudan

**Keywords:** age, ICSI, intracytoplasmic sperm injection, infertility, Sudan

## Abstract

*Background*

Intracytoplasmic sperm injection (ICSI) was considered as the mainstay of treatment for male infertility. Nowadays, the scope of ICSI has been widened to include other causes of infertility. There are few published data on ICSI in countries with low incomes.

*Aims*

A cross-sectional study was conducted at Saad AbuAlla and Banoun Centers, Khartoum, Sudan to investigate outcomes of ICSI and to determine the parameters that might predict pregnancy success rate following ICSI.

*Methods*

The study included 191 infertile couples who underwent 296 ICSI cycles between 1st April 2013 and 31 March 2014.

*Results*

One hundred and ninety one couples (comprising 296 cycles of ICSI) were enrolled to the study. The mean (SD) number of retrieved oocytes was 9.7 (7.5).  The mean (SD) number of transferred embryos was 2.9 (1.0). Out of these, 50 (26.2%) and 40 (20.9%) had chemical and clinical pregnancy, respectively. Thirty–six couples (18.8%) and five couples (2.6%) had miscarriage and had ectopic pregnancy, respectively. Under logistic regression, younger age (OR = 0.8, 95% CI= 0.81 ─ 0.96, P = 0.004) and endometrial thickness (OR = 1.3, 95% CI= 1.07─1.60, P = 0.009) were the significant predictors for the success of ICSI in inducing pregnancy.

*Conclusion*

The rates of successful fertilisation and pregnancy-to-term rates in this setting depend mainly on the maternal age.

## Introduction

*In vitro* fertilization (IVF) is recognized as the last treatment option for infertile couples who want biological children, and has been widely accepted as the most important and efficient treatment for infertility ( Khalaf *et al.*, 2008). Intracytoplasmic sperm injection (ICSI) is the gold-standard technique for the treatment of male factor infertility (Oehninger *et al.*, 2002). However, ICSI or IVF is also recommended to patients with tubal factor infertility ( Staessen *et al.*, 1999), as well as treatment of infertile couples with unexplained infertility and some polycystic ovary syndrome (PCOS) cases ( Van der Westerlaken *et al.*, 2005; Youn *et al.*, 2011). Unfortunately due to the high cost, IVF/ICSI services are not widely available at both public and private health institutions in developing countries ( ESHRE, 2008). However, in countries with lower incomes, the utility of infertility treatment is not well-established and there are few existing private IVF/ICSI centers, and those that exist are associated with a high cost; beyond the reach of most couples ( Giwa-Osagie, 2004; Otubu *et al.*, 2006). Because ICSI has a high cost to both the treatment-seeking couple and the health care system, it is necessary to assess its efficacy in different settings. Research in the IVF/ICSI field is of importance for both the treating physicians and the healthcare policy makers and will yield data necessary for patients' counseling. Different success rates/outcomes of ICSI have also been observed in different settings. There are few published data on the outcome of ICSI in countries with low income and there is no published data on ICSI in Sudan. The current study was conducted at Khartoum, Sudan to investigate ICSI outcome and to determine the parameters that might predict pregnancy success rate resulting from ICSI. Different causes of infertility, and both male and female infertility were observed in Sudan ( Elussein *et al.*, 2008).

## Methods

A cross-sectional study was conducted during the period of 1st April 2013 through to 31 March 2014 at Saad AbuAlla and Banoun Centers, Khartoum, Sudan to investigate ICSI outcome and to determine the parameters that might predict pregnancy success rate following ICSI.

After signing an informed consent form, a questionnaire was used to gather information about age, parity, menstrual history, duration of infertility, type of infertility (male infertility, failure of ovulation, tubal infertility, unexplained infertility, endometriosis and PCO), cause of infertility, number of previous cycle, endometrial thickness, number of embryos transferred, and the outcome of ISCI (pregnancy rate, rate of miscarriage and ectopic pregnancy).

Couples where males had testicular atrophy, and/or females had uterine anatomical abnormalities, were aged > 44 years, had experience uterine fibroids and/or ICSI failure more than three times were excluded from the study.

In female participants, follicle-stimulating hormone (FSH) and luteinizing hormone (LH) were measured on day 3 of the cycle; preceding ovarian stimulation which was performed followed the short GnRH agonist protocol ( Ergenoğlu *et al.*, 2012).

After the workup was done (physical examination, blood group, complete hemogram, viral screening for HIV, HBV and HCV) in the previous cycles, pituitary down-regulation started on the second day of the cycle by daily subcutaneous injection of gonadotrophins and continued until ≥ 3 follicles were present that measured ≥ 17 mm when a 10,000 IU dose of human chorionic gonadotrophin (hCG) was given. Oocyte pickup was scheduled 34–35 hours after the hCG injection. The dose of hCG ranged between 150–450 IU, depending upon the patient's age, and in response to ovarian stimulation in previous ICSI procedures. Transvaginal ultrasound was done on the day of stimulation to exclude ovarian cysts, and on cycle day seven and every other day to monitor follicle size. E2 (17 beta-estradiol) level was measured on cycle day two and when follicle maturation was achieved. In poor respondents, stimulation was stopped at 20th day of the cycle.

Ovum pickup was done under general anesthesia using a laryngeal mask airway using propofol lipuro 1% (10 mg/ml) 20 ml IV, plus atropine 0.5 mg IV, plus 4 mg dexamethasone as needed to prevent laryngeal spasm, in addition to the anesthetic gas, Nitrous oxide. Fentanyl IV was given as analgesic. Follicles were flushed using flush media from Origio (SynVitro™ Flush, Denmark) using a double lumen needle from (Origio®) if the number of follicles was ≤ 5; otherwise, a single lumen needle from (Wallace®, Wallace Ltd, Colchester, England) was used, without flushing. Embryo transfer was done without anesthesia or sedation using a soft catheter from (Wallace®). Briefly, under sterile condition, vaginal parts were cleaned with saline and draped and a Cusco speculum inserted to expose cervix. Cervical mucus was aspirated. The embryos were deposited in uterine cavity under ultrasound guidance at a position approximately 1cm shorter than the fundus. The catheter was then checked under a dissecting microscope for retained embryos. If these were found, they were reloaded and transferred again (repeat transfer). The patients were asked to remain in bed for 15–30 min following transfer.

## Statistics

The data were entered into computer using SPSS for Windows version 16.0. The mean (SD) of the ICSI variables (age and BMI) were compared between the women who had clinical pregnancy and women who had not using a Student’s t-test. These variables were compared between the different age groups using one–way ANOVA for continuous variables and Pearson’s chi-squared (X ^2^) test for the proportions of the pregnancy rate, ectopic pregnancies and miscarriage. Logistic regression was performed where induction of clinical pregnancy was the dependent variable and the ICSI variables (age, type and duration of infertility, endometrial thickness and the number of oocytes retrieved and their stage of maturation) were the independent variables. A P value < 0.05 was considered significant.

## Ethics

The study received ethical clearance from the Research Board at Department of Obstetrics and Gynecology, Faculty of Medicine, University of Khartoum, Sudan.

## Results

One-hundred and ninety-one couples were enrolled to the study, comprising 296 total cycles of ICSI. Out of these 191 couples; 82 (42.9%), 48 (25.1%), seven (3.7%) and 54 (28.3%) had male, female, combined and unexplained infertility, respectively. The vast majority (160; 83.8%) of these 191 couples had primary infertility (failure to achieve pregnancy after one year of unprotected intercourse) and the rest (31; 16.2%) had secondary infertility (failure to achieve pregnancy after one year of unprotected intercourse with previous pregnancy(ies) regardless of its outcome). The mean (SD) duration of infertility was 6.6 (4.4) years. Maternal age range was 18–44 years and the mean (SD) was 32.7 (6.2) years.

**Figure 1.**
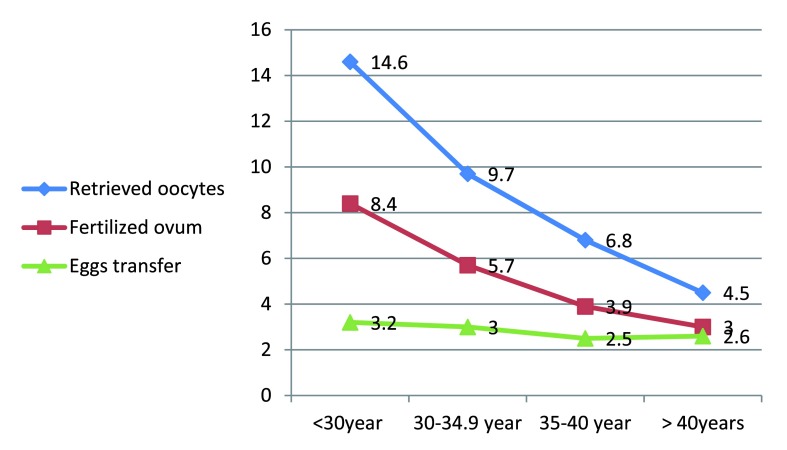
The number of the retrieved, fertilized oocytes, the transferred embryos and maternal age.

The mean (SD) number of retrieved oocytes was 9.7 (7.5). The mean (SD) number of transferred embryos was 2.9 (1.0). The number of retrieved, fertilized oocytes and the transferred embryos was significantly higher in women with age < 30 years ( Table 1; Figure 1).

**Table 1.** Comparison of different parameters between different age groups of ICSI at Khartoum, Sudan.

Variable	<30 year (n=55)	30–34.9 year (n=60)	35–40 year (n=54)	>40 years (n=22)	P
Body mass index, K/m ^2^	26.9(5.8)	29.0(6.5)	29.5(4.5)	31.3(5.8)	0.016
Follicle stimulating hormone, IU/L	6.3(2.1)	7.3(3.2)	8.1(3.6)	9.5(3.6)	<0.001
Luteinizing hormone, IU/L	4.7(2.5)	5.2(24)	4.2(2.6)	4.4(3.6)	0.762
Days of stimulation	11.1(2.1)	10.8(1.8)	11.3(1.6)	11.7(2.9)	0.298
Endometrial thickness, mm	10.5(1.5)	10.5(1.9)	10.1(1.8)	10.0(1.6)	0.462
Eggs retrieved	14.6(8.3)	9.7(6.3)	6.8(6.2)	4.5(2.5)	<0.001
Fertilized ovum	8.4(6.3)	5.7(4.5)	3.9(3.4)	3.0(1.5)	<0.001
Egg transfer	3.2(0.8)	3.0(0.9)	2.5(1.0)	2.6(1.1)	<0.001

**Figure 2.**
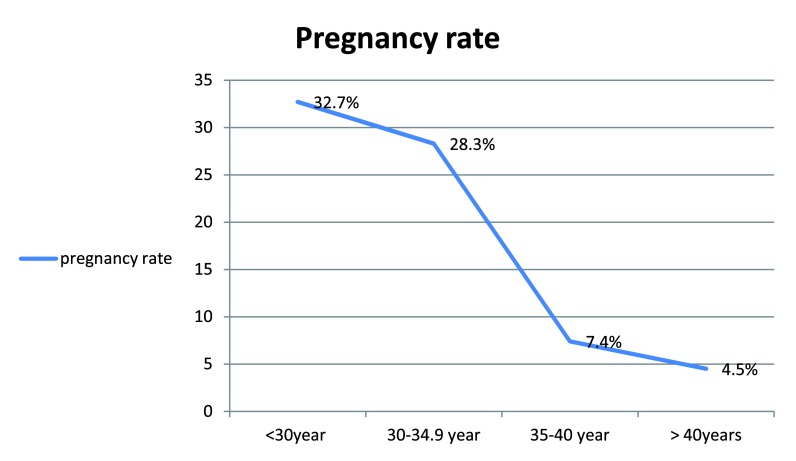
Pregnancy rate and maternal age.

Out of these 50 (26.2%) and 40 (20.9%) had chemical and clinical pregnancy, respectively. Thirty-six (18.8%) and five (2.6%) had miscarriage and ectopic pregnancy, respectively. The rate of induction of pregnancy was significantly higher in women of < 30 years of age ( Table 2; Figure 2).

**Table 2.** Age groups and the outcomes of ICSI among women at Khartoum, Sudan.

Variable	<30 year (n=55)	30–34.9 year (n=60)	35–40 year (n=54)	>40 years (n=22)	P
Miscarriage	9(16.4)	11(18.3)	10(18.5)	6(27.3)	0.739
Ectopic	2(3.6)	3(5.0)	0(0)	0(0)	0.308
Pregnancy	18(32.7)	17(28.3)	4(7.4)	1(4.5)	0.001

While the mean (SD) of the age [29.8 (4.7) vs. 33.5 (6.3) years, P = 0.001] was significantly higher, the endometrial thickness [11.1 (2.2) vs. 10.2 (1.7) mm, P = 0.005] was significantly higher in the women with clinical pregnancy (n=40) than in women who had no pregnancy (n=151). Seventeen (42.05%) out of the 40 couples who experienced successful ICSI had male factor infertility, whereas 65 couples (43.0%; P = 0.767) in which ICSI were unsuccessful had male factor infertility ( Table 3).

**Table 3.** Comparison of various variables between pregnant and non-pregnant women following ICSI at Khartoum, Sudan.

Variable	Pregnancy (n=40)	No pregnancy (n=151)	P
Age, year	29.8(4.7)	33.5(6.3)	0.001
Body mass index, kg/cm ^2^	28.6(6.6)	28.8(5.7)	0.825
Duration of infertility, years	5.8(3.1)	6.9(4.7)	0.174
Follicle stimulating hormone, IU/l	7.2(3.0)	7.6(3.3)	0.501
Luteinizing hormone, IU/l	5.8(9.1)	4.4(2.7)	0.109
Days of stimulation	11.4(2.4)	11.1(1.9)	0.354
Endometrial thickness, mm	11.5(2.2)	10.2(1.7)	0.005
17 beta-estradiol at triggering, Pg/ml	3326(2710)	2878(2379)	0.305
Eggs collected	11.6(8.0)	9.3(7.3)	0.082
Fertilized ovum	7.2(5.6)	5.3(4.7)	0.028
Egg transfer,	3.2(0.7)	2.8(1.0)	0.016
Days of transfer	3.9(0.8)	3.7(0.8)	0.139

In logistic regression, younger age (OR = 0.8, 95% CI = 0.81–0.96, P = 0.004) and endometrial thickness (OR = 1.3, 95% CI = 1.07–1.60, P = 0.009) were the significant predictors for the success of ICSI treatment ( Table 4). Raw dataset available in Dataset 1.

**Table 4.** Logistic regression of the predictors of pregnancy following ICSI at Khartoum, Sudan.

Variables	OR	95% CI	P
Age	0.8	0.81–0.96	0.004
Body mass index	1.0	0.97–1.11	0.264
Male infertility	0.6	0.23–1.58	0.306
Follicle stimulating hormone	0.9	0.83–1.13	0.762
Luteinizing hormone	1.0	0.96–1.16	0.205
Estradiol levels	1.0	1.00–1.00	0.864
Endometrial thickness	1.3	1.07–1.60	0.009
Type of the catheter	2.532	0.195–2.888	0.478

Raw data for Ahmed *et al.*, 2015 'Maternal age and intracytoplasmic sperm injection outcome in infertile couples at Khartoum, Sudan' Age= maternal age (years). BMI= Body mass index (maternal, Kg/m ^2^). FSH= Follicle stimulating hormone, IU/l. LH= Luteinizing hormone, IU/l. Factor= infertility factor: 1= male, 2= tubal, 3= ovarian, 4= combined, 5= unexplained. Protocol= Protocol used (1= long, 2= short, 3= antagonist, 4= agonist –antagonist, 5= short soft). Days= days of stimulation. Endometrium= Endometrial thickness (mm). E2= 17 beta-estradiol at triggering, (Pg/ml). EC= eggs collected. M2= eggs at metaphase 2. Fertilized= no. of fertilized eggs. DOT= day of transfer. ET= eggs transferred. Chempreg= chemical pregnancy (0= no, 1= Yes). Clinpreg= clinical pregnancy (0= no, 1= yes). Catheter= catheter type. Duration of infertility= duration of infertility (years). Primsec= type of infertility (0= primary infertility, 1= secondary infertility). Miscarriages= miscarriage (0= no, 1= yes). Ectopic pregnancies= ectopic pregnancy (0= no, 1= yes). Age group (0= less than thirty years, 1= thirty-thirty five years, 2= thirty five – forty years, 3= More than forty years) ( Ahmed *et al.,* 2015). Click here for additional data file. Copyright: © 2015 Ahmed M et al. 2015 Data associated with the article are available under the terms of the Creative Commons Zero "No rights reserved" data waiver (CC0 1.0 Public domain dedication).

## Discussion and conclusions

The main findings of the current study were that the number of eggs retrieved, fertilized ovum, the number of embryos successfully transferred and the rate of successful induction of pregnancy depend on age of the woman and endometrial thickness. The pregnancy rate (20.9%) in this study was lower than the rates recently reported in Nigeria (30%; Orhue *et al.*, 2012); Tunisia (32.4%; Fourati *et al.*, 2009), Vienna, Austria (27.3%; Nouri *et al.*, 2015) and in Singapore ( Tan *et al.*, 2014). It is worth mentioning, however, that all of these studies (with exception of Fourati *et al.*) report the pregnancy rate following IVF/ICSI and not the rate following ICSI alone, as in our study.

In the current study, ICSI outcomes such as eggs retrieved, fertilized ovums, embryos transferred and the rate of successful induction of pregnancy depend primarily on age of the woman, where the optimal outcomes were observed in women < 30 years of age. This is consistent with Tan *et al.*’s (2014) findings where optimal IVF outcomes (the number of oocytes retrieved) was highest among women aged < 30 years, with a reduced number of oocytes retrieved per cycle, lower pregnancy and live birth rates seen among women of older age groups. Likewise, Nouri *et al.* (2015) observed that age was an independent factor for pregnancy rate following IVF/ICSI. The decreasing ovarian reserve ( Speroff, 1994), poor oocyte quality ( Simpson *et al.*, 2000), higher embryo implantation failure ( Navot *et al.*, 1991), ovulatory dysfunction due to poor hormonal environment ( Hull *et al.*, 1996; Sherman *et al.*, 1976) and uterine problems ( Faddy *et al.*, 1992; Scwartz & Mayaux, 1982) were the postulated effects of the aging process that could have a detrimental effect on the efficacy of IVF/ICSI.

In the current study the pregnancy rate was associated with endometrial thickness. This agrees with the several previous studies which have shown a significant correlation between pregnancy rate and endometrial thickness ( Al-Ghamdi *et al.,* 2008; Kasius *et al.*, 2014; Okohue *et al.*, 2009). Endometrial thickness <7 mm was reported to have a significant reduction in the implantation rate and pregnancy rate. It has recently been shown that (systematic review and meta-analysis) probability of clinical pregnancy for an endometrial thickness ≤7 mm was significantly lower compared with cases with endometrial thickness >7 mm which investigated for pregnancy outcomes after IVF ( Kasius *et al.*, 2014).

We conclude that the fertilization and pregnancy rates in this setting depend mainly on maternal age.

## Data availability

The data referenced by this article are under copyright with the following copyright statement: Copyright: © 2015 Ahmed M et al.

Data associated with the article are available under the terms of the Creative Commons Zero "No rights reserved" data waiver (CC0 1.0 Public domain dedication). 

*F1000Research*: Dataset 1. Raw data for Ahmed *et al.*, 2015 'Maternal age and intracytoplasmic sperm injection outcome in infertile couples at Khartoum, Sudan', 10.5256/f1000research.7386.d107727

## Supplementary materials

**Questionnaire for collection of ICSI data.**

After signing an informed consent form, the questionnaire was used to gather information about age, parity, menstrual history, duration of infertility, type of infertility (male infertility, failure of ovulation, tubal infertility, unexplained infertility, endometriosis and PCO), cause of infertility, number of previous cycle, endometrial thickness, number of embryos transferred, and the outcome of ISCI (pregnancy rate, rate of miscarriage and ectopic pregnancy).
