# Supplementary material for: Maternal age and intracytoplasmic sperm injection outcome in infertile couples at Khartoum, Sudan
Source: F1000Res. 2015 Nov 24;4:1339. [Version 1] doi: 10.12688/f1000research.7386.1 (PMC4909122; doi:10.12688/f1000research.7386.1)
Supplement: Supplementary file 2 [file f1000research-4-7960-s0001.tgz › 585c0683-8592-49b2-bdcc-e34fa5f3f2f0.docx]

**Questionnaire (English) for ICSI data collection**

Age------type of infertility: primary------secondary

Factor of infertility: male- --tubal------ovarian---------combined-----unexplained-----

Weight in Kg---------Ht in cm--------

LH------ FSH------- estradiol level-----

Endometrial thickness---------

Egg retrieved----egg fertilized----------- embryo transferred-------------

Day of transfer -------

Type of the Cather: labotect----m.walace----coak

Chemical pregnancy no-------yes------- clinical pregnancy no----- yes------

Misarrange: no----- yes-------ectopic pregnancy no------ yes-------

others ---------------

--------------------------
