# Supplementary material for: Maternal age and intracytoplasmic sperm injection outcome in infertile couples at Khartoum, Sudan
Source: F1000Res. 2015 Nov 24;4:1339. [Version 1] doi: 10.12688/f1000research.7386.1 (PMC4909122; doi:10.12688/f1000research.7386.1)
Supplement: Peer review history

# Peer review: Referee response for version 1

**DOI:** 10.5256/f1000research.7960.r14119

**Published:** 2016-06-14

**Authors:** Silke J Dyer, Paversan Archary

**Affiliations:** 1 Department of Obstetrics & Gynaecology, University of Cape Town, Cape Town, South Africa

Thank you for giving us the opportunity to review this article. The authors report on female factors impacting outcome and pregnancy success rate following ICSI for treatment of infertility in a low income setting.

 We congratulate and thank the authors on publishing findings on the effectiveness of ART in their country, as data pertaining to the availability, utilization, effectiveness and safety of ART in sub-Saharan Africa are overall scant and much needed.

 The paper is generally well written. Data are presented clearly, and the tables and illustrations are relevant. The study design is appropriate, and the analysis and interpretation of data are overall sound. The conclusions derived are balanced and justified. Although the main finding, especially in relation to age is not novel, the paper contributes relevant information on the status of ART in Sudan. 

 The paper could be further strengthened if (1) information on biochemical pregnancies was excluded and simply considered under “not pregnant”; (2) data on live births and on complications of ART especially multiple pregnancies were included or, if not available, acknowledged as a study limitation; (3) more information on semen quality and prevalence of severe male factor infertility in the study group were presented; (4) a section on study strengths and limitations was included. 

 We have the following additional major and minor observations/ suggestions in order of appearance in the manuscript:

 Major:

1. According to current evidence ICSI should be considered first line intervention for severe male factor infertility. The use for other indications is of no proven benefit according to our understanding of current evidence, although we acknowledge that it is used for other indications as indeed in the index study (see ICMART World report 2008-2009-2010 and accompanying Editorial).  The authors may wish to stay away from a discussion on the role and indications of ICSI in the introduction as in our opinion it weakens the paper; a comment under study limitations may suffice. Instead, it would be of interest to briefly expand on the current situation of ART in Sudan in the introduction.
2. In table 3, number of eggs fertilized and numbers of eggs transferred also differed between the two groups (p<0.05). It is not clear if these variables were included in the regression analysis- and if not, why. Either way it justifies some attention in both the result and discussion section. 

Minor:

1. The title is somewhat misleading and could be rephrased. Perhaps “Factors predicting pregnancy following ART with ICSI in Sudan” or similar would capture the study better?
2. The conclusion in the abstract is not strictly based on the results as no information pertaining to fertilisation are presented in the abstract.
3. Page 3 column 2; line 1 and 2 should be revised. At present the reader gets the impression that gonadotrophins rather than GnRH agonists are used for pituitary down-regulation. In ‘The dose of hCG ranged between 150 – 450IU’ presumably FSH and not hCG is meant. In addition, the entire section on methods could perhaps be shortened, at least if we assume that readers are familiar with the basic principles of ART.  
4. Presumably “pregnancy” in table 2 and figure 2 refers to “clinical pregnancies’ only. If the authors follow our recommendation to include biochemical pregnancies under “not pregnant” no changes are required; otherwise an asterix in both the table and figure is needed to specify clinical versus biochemical pregnancy.
5. The choice of age groups is not in keeping with age categories used by regional/international data registries. This limits valuable comparisons.  It would be advantageous to consider these age categories in future work.

We have read this submission. We believe that we have an appropriate level of expertise to confirm that it is of an acceptable scientific standard, however we have significant reservations, as outlined above.

## References

[1] DyerSChambersGMde MouzonJNygrenKGZegers-HochschildFMansourRIshiharaOBankerMAdamsonGD: International Committee for Monitoring Assisted Reproductive Technologies world report: Assisted Reproductive Technology 2008, 2009 and 2010. *Hum Reprod*.2016;31(7) : 10.1093/humrep/dew082 1588-609 10.1093/humrep/dew082 27207175

[2] EversJ: Santa Claus in the fertility clinic: Table I. *Human Reproduction*.2016;31(7) : 10.1093/humrep/dew092 1381-1382 10.1093/humrep/dew092 27207176

# Peer review: Referee response for version 1

**DOI:** 10.5256/f1000research.7960.r13995

**Published:** 2016-05-26

**Authors:** Akmal El-Mazny

**Affiliations:** 1 Department of Obstetrics and Gynecology, Cairo University, Cairo, Egypt

The title is appropriate for the content of the article, and the abstract represents a suitable summary of the work. The design, methods, and analysis of the results from the study have been explained and are appropriate for the study. The conclusions are sensible, balanced, and justified on the basis of the results, and enough information has been provided in a usable format to be able to replicate the study.

 Recommendations:

 Methods:

- Adequately describe inclusion and exclusion criteria.
- Mention appropriate details of ovarian stimulation and ICSI technique.

Results:

- The text should complement the tables and figures (do not duplicate).
- Clarify the effect size for the main outcome.

Discussion:

- Mention the differences from other studies.
- Mention the limitations of the study.
- Suggest clinical implications.

I have read this submission. I believe that I have an appropriate level of expertise to confirm that it is of an acceptable scientific standard.

# Peer review: Referee response for version 1

**DOI:** 10.5256/f1000research.7960.r12588

**Published:** 2016-03-10

**Authors:** Frank H de Jong

**Affiliations:** 1 Department of Internal Medicine, Erasmus University Medical Center, Rotterdam, 3000 CA, Netherlands

In this manuscript, results of an ICSI programme in Khartoum, Sudan are reported. The amount of new information is limited: as indicated in the discussion, a host of published papers already concluded that maternal age is an important factor determining the success of ICSI or IVF procedures.

The manuscript might be improved on the following aspects:

1. There is hardly information on the male partners. Data on hormone levels and sperm quality might be as important as the data on hormone levels, endometrial thickness and age in the female partners.
2. There is no information on sperm retrieval, ICSI procedure or embryo culture.
3. On page 3, right-hand column, line 6 "HCG"should probably read "FSH".
4. What happens when pregnancy results are normalized for numbers of embryos transferred? Can the difference in numbers of pregnancies in the different age groups be explained on basis of this number?
5. It might be better to replace "ovum" (line 2 under Discussion and Conclusions) and "ovums" (line 2 in right-hand column on page 6) with "ova".

I have read this submission. I believe that I have an appropriate level of expertise to confirm that it is of an acceptable scientific standard, however I have significant reservations, as outlined above.
